# Physiochemical properties of Sarawak's adapted Liberica coffee silverskin utilizing varying solvents

**DOI:** 10.1002/fsn3.3541

**Published:** 2023-07-06

**Authors:** Nick Laurence Buyong, Elexson Nillian

**Affiliations:** ^1^ Faculty of Resource Science and Technology University Malaysia Sarawak Kota Samarahan Sarawak Malaysia

**Keywords:** antioxidant activities, coffee by‐product, total flavonoid content, total phenolic content

## Abstract

This study aimed to investigate the physiochemical properties of Sarawak's adapted Liberica coffee silverskin (CS) using multiple solvents (distilled water, methanol, and ethanol) and its impact on the total phenolic content (TPC), total flavonoid content (TFC), and antioxidant activities of the CS. The results showed that the highest TPC was observed in the methanol extract (15.24 ± 0.65 mg GAE/g), while the highest TFC was recorded when extracted with ethanol (25.14 ± 0.59 mg QE/g). The DPPH activity was also found to be highest in the ethanol extract (83.85 ± 1.78%), concurred by the results in the FRAP assay as the highest reduction was also in ethanol (11.40 ± 18.57 μmol FSE/g). These findings demonstrate that the bioactive compounds of CS extracted can be greatly influenced by the choice of solvent while highlighting the potential for Sarawak's adapted Liberica CS to be further harnessed into a value‐added product and enabling a better by‐product waste management.

## Graphical abstract

This study focused on the Liberica coffee silverskin (CS) indigenous to the land of Malaysian state of Sarawak. The CS was found to be high in phenolic content, flavonoid content, and antioxidant activity. Even with different solvents used for extraction, the Liberica CS shows promising beneficial phenolic compounds for further utilization in the coffee market.

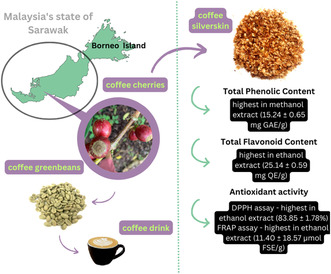

## 1 INTRODUCTION

Liberica is a rare variety of coffee, and it is grown in small quantity compared to Arabica and Robusta. This species accounts for 73% of all coffee cultivation in Malaysia. Robusta coffee comes in second with a 27% coverage, and both are suited for cultivation in Malaysia due to their favorable climate (Ismail et al., 2014). In Malaysia alone, the total weight of coffee consumption recently in 2022 was 800,000 of 60 kg bags which brings to a total of 48,000 metric tons of coffee (Ahmad, 2022). According to a newspaper report by The Star, Liberica coffee production was at approximately 325,584 kg in June 2022 (Benjamin, 2022). The differences in the demand and supply of coffee beans make Malaysia not only as a producer but also as one of the players in import and export of coffee from neighboring countries such as Indonesia and Vietnam, some even from Brazil. In comparison with Singapore, the third major importer of coffee beans worldwide, Malaysia's international trade of shipments is in the ratio of 1:6 to Singapore (Volza Grow Global, 2023). However, this is due to Malaysia only imports to supplement the coffee supply and justifies consumer demands. Malaysia is no powerhouse of coffee compared to countries such as Brazil, Panama, and Colombia, the three main exporters of coffee. Despite that, Malaysia is one of the main Liberica coffee cultivators in Southeast Asia alongside the Philippines (Anindya, 2021).

In terms of coffee bean production, the Liberica generated a lower amount of green beans (0.7:10) in kilograms compared to Arabica and Robusta (2:10) in kilograms, by being scarcely available in the market, the Liberica variety serves a higher value in the world coffee market compared to the other species (Yoong, 2022). As one of the main manipulators of the Liberica coffee, Malaysia plays an important part in ensuring the sustainability of coffee variety in the world coffee market (Ali & Ramanathan, 2021), while possibly benefiting from the high revenue stream. Not to mention the fact of a warmer climate, Liberica could re‐emerge as a major coffee species due to its robust growth habit and ability to grow in warm, and lowland. This has been displayed before as the Liberica variety was able to survive the coffee leaf rust disease in the early 19th century compared to the other varieties (Davis et al., 2022).

During roasting of coffee beans, the heat generated will result in the production of a thin tegument known as the coffee silverskin (CS) (Bessada et al., 2018). CS is a by‐product of coffee production, and it is often conventionally discarded as waste. Other than that, currently, the CS also undergoes extraction and encapsulation of the bioactive compounds to be incorporated into various foods and nonfood purposes (Tores de la Cruz et al., 2019). A study by Rodrigues et al. (2015) supports that CS has a high content of cellulose, hemicellulose, and lignin, which makes it a potential source for biofuel production. The CS only constituted 4% (w/w) of the coffee cherry (Martuscelli et al., 2021), but in the production of 1 ton of coffee, approximately 40 kg of CS will be produced, with the production of tons of thousands of coffee per year, the CS also produced in excess which can cause high environmental pollution with an improper waste management. From a bird's eye view, this study can give an insight into the potential health benefits of Liberica coffee, while contributing to the scarce literature on Liberica coffee and waste by‐product.

To our knowledge, there are no research currently on Liberica coffee that are native to the land of Sarawak, Malaysia. There are some studies done on silverskin, but the majority of them are focused on other species such as Arabica and Robusta instead of Liberica. One of the recent studies showed a promising result as there are high antioxidant contents and high total phenolic content but with low toxic mineral level such as nickel found in Arabica CS and Robusta CS (Martuscelli et al., 2021) In the coming years, more papers on silverskin emerging (Ballesteros et al., 2014; Costa et al., 2018; Vimercati et al., 2022), but less directed on the Liberica variety. Thus, the aim of this study is to perform a novel investigation on the physiochemical properties of Sarawak's Liberica CS using multiple different solvents, and its impact on the total phenolic and flavonoid content, and the antioxidant activities of the CS extract. Thus, opening multiple potentials for it to become a value‐added product.

## 2 MATERIALS AND METHODS

### 2.1 Sample preparation

In collaboration with coffee industries, the Sarawak Liberica CS sample was obtained from RekaJaya Plantation Sdn Bhd, an industrial partner in Kuching, Sarawak. The Liberica green beans were submitted for roasting at a temperature of 220°C for 15 min to obtain the by‐product CS. Liberica CS was collected and ground using a basic blender to break down the CS further into smaller sizes (1–10 mm) in diameter to maximize extraction. One gram of CS was macerated with 20 mL of solvents (methanol (99.8%), ethanol (95%), and distilled water) inside a 50‐mL conical flask, respectively, the mixture was then inserted into the incubator shaker for 30 min at 60–65°C. Whatman No. 1 paper filters were used to filter the extracts. The extract was then stored at 3–5°C inside an amber vial for further analysis. The concentration of the extracts was made to 1 mg/mL (w/v) with respective solvents for analysis (Ballesteros et al., 2014).

### 2.2 Total phenolic content

**FIGURE 1:**
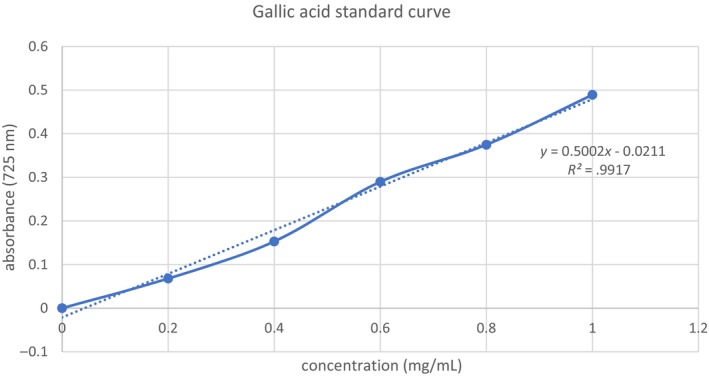
Gallic acid standard curve with concentrations tested 0.2–1.0 mg/mL.

The Folin–Ciocalteu technique (Utami et al., 2013) was used to estimate the TPC. In a nutshell, 0.75 mL of the Folin–Ciocalteu reagent was combined with 0.1 mL of the extract (3 mg/mL) and allowed to react for 5 min. Following the addition of 0.75 mL of Na_2_CO_3_ 6% (w/v) solution, the mixture was left for 90 min in the dark and at room temperature. The absorbance at 725 nm was measured using a spectrophotometer. The procedure was performed in triplicate to obtain a mean reading for significant analysis. The calibration curve for gallic acid obtained was *R* ^2^ = .998 within a linearity range of 0.2–1.0 mg/mL (Refer to Figure 1). The results were expressed as milligram of gallic acid equivalents per gram of CS (mg GAE/g).

### 2.3 Total flavonoid content

**FIGURE 2:**
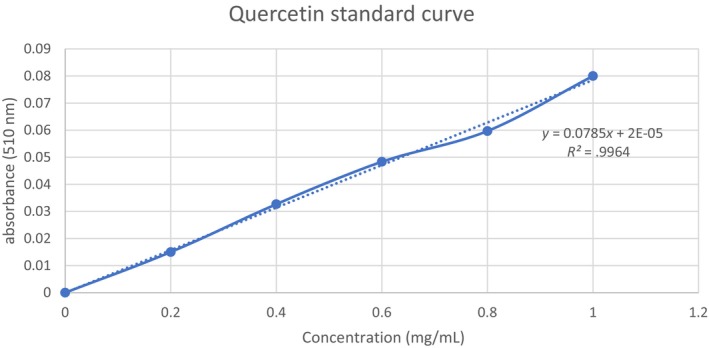
Quercetin standard curve with concentrations tested 0.2–1.0 mg/mL.

According to Rodrigues et al. (2015), TFC can be measured using the colorimetric assay. In brief, 0.75 mL of NaNO_3_ solution (5% w/v) was mixed with 0.125 mL of extract (1 mg/mL), and the mixture was left for 6 min in the dark and at room temperature. The mixture was then added with 0.150 mL of 10% AlCl_3_. The final volume of 2.5 mL was then reached by adding 0.750 mL of NaOH (1 M) and 1.4 mL of distilled water into the mixture. After 5 min of incubation in the dark and at room temperature, the absorbance of the mixture was then measured at 510 nm. A quercetin calibration curve was employed with an *R* ^2^ = .99 and a linearity range of 0.2–1.0 mg/mL (Refer to Figure 2). The results were reported as milligram of quercetin equivalents per gram of dried CS weight (mg QE/g).

### 2.4 2,2‐Diphenyl‐1‐piccrCylhydrazyl (DPPH) assay

**FIGURE 3:**
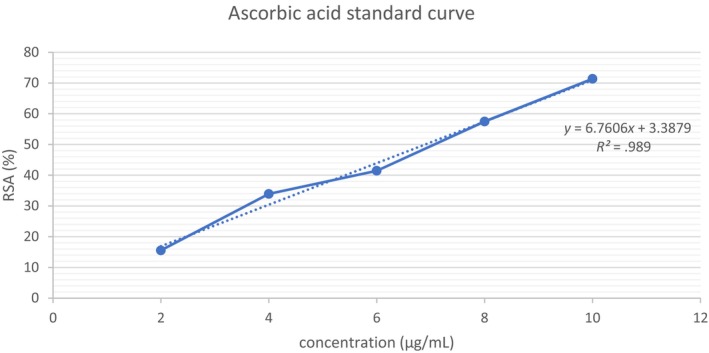
Ascorbic acid standard curve with concentrations ranging from 2 to 10 μg/mL.

DPPH's radical‐scavenging activity (RSA) was used to measure the extracts' antioxidant activities. A 1.0 mL of DPPH solution was added into a range of concentration of the sample (0.2–1.0 mg/mL). After 35 min in the dark, the absorption was read at 515 nm (Rodrigues et al., 2015). A calibration curve of ascorbic acid with a linearity range of 2–10 μg/mL and *R* ^2^ = .99 was prepared (Refer to Figure 3), while the inhibitory concentration needed to decrease the initial DPPH by 50% (IC_50_) was obtained from the linear equation obtained. The results were expressed as microgram of ascorbic acid equivalent per gram of sample (mg AAE/g). The RSA was calculated in percentage according to the following equation:

$$\mathrm{RSA}\%=\frac{{\mathrm{Abs}}_{\text{control}}-{\mathrm{Abs}}_{\text{sample}}}{{\mathrm{Asb}}_{\text{control}}}\times 100$$

Where ${\mathrm{Abs}}_{\text{control}}$ is the control's absorbance reading, while ${\mathrm{Abs}}_{\text{sample}}$ is the sample's absorbance reading at 515 nm, respectively.

As for the DPPH value, the calculation of the ascorbic acid equivalent per gram of the sample (mg AAE/g) was done by the following equation:

$$\text{DPPH value}\;\left(\text{mg AAE}/g\right)=\left(\frac{{\mathrm{IC}}_{50}\left(\text{control}\right)}{{\mathrm{IC}}_{50}\left(\text{sample}\right)}\right)\times \left(\frac{C}{W}\right)$$

Where IC_50_ can be obtained from the linear equation generated by the RSA against concentration graph, *C* is the initial concentration (mg/mL) of the control (ascorbic acid), and *W* is the dry weight (g) of the CS extract.

### 2.5 Ferric reducing antioxidant power (FRAP) assay

**FIGURE 4:**
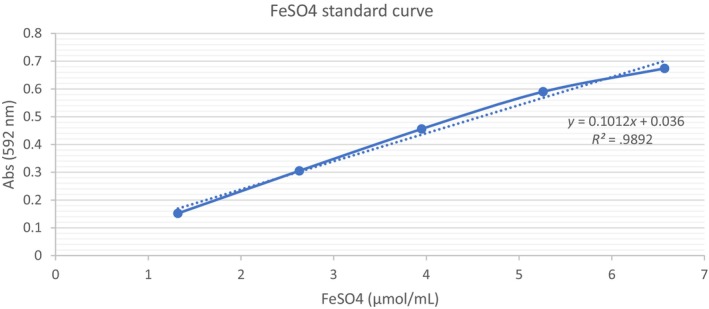
Ferrous sulfate standard curve with concentrations tested 1.32–6.57 μmol/mL.

For the FRAP assay, according to Benzie and Strain (1996), 3 mL of the FRAP reagent (10:1:1 of 300 mM sodium acetate buffer at pH 3.6, 10 mM TPTZ, and 20 mM FeCl_3_) was combined with 0.1 mL of a range of sample concentrations (0.2–1.0 mg/mL), the mixture was then incubated at 37°C for 20 min. The increase in absorbance at 592 nm was read using a spectrophotometer. It was calibrated using a FeSO_4_ with linearity range of 1.32–6.57 μmol/mL and *R* ^2^ = .99 (Refer to Figure 4). The results were expressed in terms of micromole ferrous sulfate equivalents per gram of sample (μmol FES/g). The FRAP value was calculated according to the following equation:

$$\text{FRAP value}\;\left(\text{μmol}\;\mathrm{FES}/g\right)=C\times V\times \frac{t}{m}$$

Where *C* is the FeSO_4_ concentration (μmol/mL) of the corresponding standard curve of the CS sample, *V* is the CS sample volume (mL), *t* is the dilution factor, and *m* is the weight of the CS dry matter (g).

### 2.6 Data analysis

The data were collected and analyzed using the Excel for Microsoft Office 365, version 2021. In this study, the data were performed and collected in triplicate to obtain the mean average and distribution of the data deviation. The one‐way ANOVA test was performed on the data to test the significance of the results.

## 3 RESULTS AND DISCUSSIONS

### 3.1 Total phenolic and flavonoid content

Based on Table 1, the methanol extract showed an exceptionally high amount of phenolic (15.24 ± 0.65 mg GAE/g). This is then followed by ethanol (11.2 ± 0.12 mg GAE/g) and water (9.48 ± 0.32 mg GAE/g) extracts. The results obtained from this study comply with the results from the study done by Nzekoue et al. (2020) on CS extraction to quantify the bioactive compounds, the solvent ratio of ethanol to water (70:30) was employed and TPC obtained was as high as 40.4 to 73.4 mg GAE/g. Even though this study achieved lower phenolic compounds compared to the previous study, this study still proved the presence of phenolic compounds in the Liberica CS, which is linked to high antioxidant activities in result 3.2.

**TABLE 1:** Total phenolic content and total flavonoid content of CS extracts.

Solvent	TPC (mg GAE/g)	TFC (mg QE/g)
Water	9.48 ± 0.32**	6.45 ± 2.79**
Methanol	15.24 ± 0.65*	21.05 ± 4.28*
Ethanol	11.2 ± 0.12*	25.14 ± 0.59*

*Note*: All data are expressed as mean ± standard deviation. ‘**’ represents significant difference at .05, while ‘*’ represents no significant difference at .05.

The results from this study are also in agreement with the study performed by Rodrigues et al. (2015). Whereas a high TPC was observed using the organic ethanol solvent as well compared to the water, a high 35.25 ± 0.25 mg GAE/g of TPC was observed compared to water with only 1.08 ± 0.23 mg GAE/g. The difference in the TPC among the solvents was expected as the CS had shown a high amount of chlorogenic acid (CGA) in the study concluded by Siva et al. (2016). However, TPC from this study was also relatively lower when compared to its own coffee bean in a study completed previously by Nillian et al. (2020). The coffee pulp was extracted with ethanol solvent, and the team managed to obtain as high as 24.24 mg GAE/g. This was expected as coffee pulp is known to have higher content of bioactive compounds such as caffeine, melanoidins, and CGA (Geremu et al., 2016; Nillian et al., 2020), thus highlighting a higher result of TPC.

Chlorogenic acid (CGA) is the most abundant antioxidant content in fruits and fresh produces that helps to keep the fresh flavor and nutritional value of the product for a longer time, the bioactive compound solubilizes easily in organic solvent compared to an aqueous which directly effecting the resulting TPC. However, the studies performed were only on Arabica and Robusta species (Liang & Kitts, 2014) and not on Liberica, it was concluded that different species of the coffee family resulted in different content of CGA. This too supported by research completed before such that Robusta CS showed a higher value of TPC than Arabica CS, although the value of TPC also may vary due to the extraction methods (Tan et al., 2016).

As referred to Table 1, the flavonoid content of the Liberica CS was significantly (*p* < .05) highest observed by extracting with organic ethanol and methanol solvent, 25.14 ± 0.59 mg QE/g and 21.05 ± 4.28 mg QE/g, respectively, compared to extraction with aqueous distilled water (6.45 ± 2.79 mg QE/g) subsequently. The flavonoid content is deemed higher in ethanol compared to methanol and water extract. This is mostly due to the solubility characteristics of the compound's group (Costa et al., 2018; Kumar & Pandey, 2013). This is certainly one of the main factors that impacted the absorption of the flavonoid by the human body. However, the results obtained from this study were consistent with the studies before such that the total flavonoid content estimated was highest when extracted by ethanol solvent even though in different coffee species (Ballesteros et al., 2014; Costa et al., 2018; Rodrigues et al., 2015).

The current study was performed on solid–liquid extraction of the CS sample. Due to the novelty of this study on Sarawak's Liberica CS, selection of the extraction solvent concentration was tested on ground zero (95%–99.8%) without the performance of solvent dilution for the alcohol solvents. The solvents chosen for this study were distilled water, methanol, and ethanol. These solvents are selected due to their abundant availability in a standard research laboratory, while economical and suitable for the extraction of phenolics and flavonoids compound. The selection of solvents is also decided based on the Green Chemistry (GC) concept such as the solvent must be considered for reduction or elimination in products, by‐products, or reagents which are hazardous to human health and environment (Joshi & Adhikari, 2019). However, there are other nontoxic and preferred solvents for extracting the phenolic and flavonoid compounds such as acetone and ethyl acetate (Joshi & Adhikari, 2019). Despite that, alcohol solvents still perform better in phenolic and flavonoid extraction as in the study by Dirar et al. (2019), ethanolic extracts showed higher phenolic and flavonoid content extracted compared to acetone and dichloromethane (DCM). The usage of DCM is very much undesirable as this solvent has very low density, is irritable, and is more toxic compared to the water and alcohol solvents.

Despite that, further study can be suggested on the dilution of the alcohol solvent on Sarawak's Liberica CS, as a study does report that the optimum conditions for the CS extraction were using 60% of ethanol in a ratio of 35 mL per gram of the CS for 30 min at 60–65°C (Ballesteros et al., 2014). These extraction conditions do concur with this current study on the Liberica CS, as the results in 3.2 showed antioxidant activities from the Liberica CS extract. However, there are also some studies suggesting that the optimal extraction conditions would be at 40°C for 40 min (Tangguh & Kusumocahyo, 2017), while some performed extraction at 40°C for 60 min (Costa et al., 2014; Tan et al., 2016), nonetheless, there are no standard extraction conditions currently for the CS, only few conditions that were explored in previous studies. While some of these studies resulted in a high extraction yield, other variables such as the solvent selection and coffee variety also play important role in optimizing yield.

In terms of extraction method, the current study utilizes the conventional maceration method to allow reproducibility by other researchers. This method is inexpensive, quick, and does not require the usage of complex and expensive machineries. In terms of the result, the TPC value (15.24 ± 0.65 mg GAE/g) does not differ significantly while extracted with methanol solvent compared to a study before (8.94 ± 0.01 mg GAE/g) which utilizes an ultrasonic machine for extraction of the plant crude (Wen et al., 2019), even lower if pointed out. This is also concurred with the research of Tangguh and Kusumocahyo (2017), such that the conventional extraction achieved a high antioxidant activity of 68.8% compared to ultrasonic‐assisted extraction method.

Overall, this study suggests that CS may be a valuable source of phenolic compounds and flavonoids, and that extracting them using ethanol or methanol solvents may be the best approach as this gives higher output on the compounds extracted. Comprehending the contents of TPC and TFC will provide a more comprehensive understanding of utilization of the CS as value‐added products. By developing and optimizing the methods to extract these compounds, coffee industries can create a revenue stream and reduce waste created by discarding of the CS as it is one of the conventional ways of postroasting (Murthy et al., 2012). Moreover, the TPC and TFC data in this study can be employed in the agricultural fields such as to monitor the quality of the coffee plant. Utilization of the data will assist the coffee breeders or producers in the enhancement of the nutritional value of the coffee beans by selective breeding. In addition, crops with high nutritional value tend to be more resistant to environmental stressors, pests, and/or even diseases. Thus, understanding of the phenolic and flavonoid content will indirectly abet the sustainability and survival of the coffee species.

### 3.2 Evaluation of antioxidant activity

Referring to Table 2, the data showed a higher percentage of free‐radical scavenging activity of DPPH by the CS sample in ethanol extract which then follows by methanol and water extract, 83.85 ± 1.78%, 78.77 ± 0.99%, and 62.84 ± 2.98%, respectively. The CS extract was allowed to counteract the DPPH free radical by donating hydrogen at concentrations ranging from 0.2 to 1.0 mg/mL. Based on Table 2, the ${\mathrm{IC}}_{50}$ was calculated from the regression line of RSA (%) against concentration (mg/mL) of each sample. The ${\mathrm{IC}}_{50}$ of the CS extract in ethanol solvent is the lowest with 0.40 ± 0.004 mg/mL. This is followed by methanol and lastly water extract with 0.59 ± 0.01 and 0.81 ± 0.03 mg/mL, respectively. The DPPH scavenging activity in this study was correlated directly proportional to the antioxidant of the sample.

**TABLE 2:** Antioxidant activity of CS extracts.

Sample	RSA (%)	IC_50_ (mg/mL)	DPPH value (mg AAE/g)	FRAP value (μmol FSE/g)
Water	62.84 ± 2.98**	0.81 ± 0.03**	0.78 ± 0.03**	2.50 ± 3.98**
Methanol	78.77 ± 0.99*	0.59 ± 0.01*	1.81 ± 0.03*	11.14 ± 17.83*
Ethanol	83.85 ± 1.78*	0.40 ± 0.004*	2.60 ± 0.02*	11.40 ± 18.57*

*Note*: All data are expressed as mean ± standard deviation. ‘**’ represents a significant difference at .05, while ‘*’ represents no significant difference at .05.

The results obtained agree with the past studies (Ballesteros et al., 2014; Costa et al., 2018), especially on the ability of the organic solvent to extract antioxidant compounds from natural sources. Other than that, the RSA obtained in this study is also in agreement with the study done by Nzekoue et al. (2020), such that the ${\mathrm{IC}}_{50}$ of the DPPH scavenging was the highest in methanol extract with 101.7 ± 5.5 μg/mL and the lowest was in water extract 362.1 ± 65.7 μg/mL. This is also predicted as the DPPH are known to have hydrophobic properties and thus are less soluble in aqueous solution compared to organic solution (Costa et al., 2018). As the CS is reported to have high number of bioactive compounds especially due to the roasting process regardless of the species, the synergistic manner of the main bioactive compounds may be responsible for this scavenging activity of the DPPH free radicals. However, as it was suggested, there is no evidence to show the correlation between the DPPH value with the TPC of the CS extracts. Thus, this suggests that the antioxidant of the CS may also come from the activities of the melanoidins and diterpenes and not entirely depend on the CGA and caffeine (Nzekoue et al., 2020).

**FIGURE 5:**
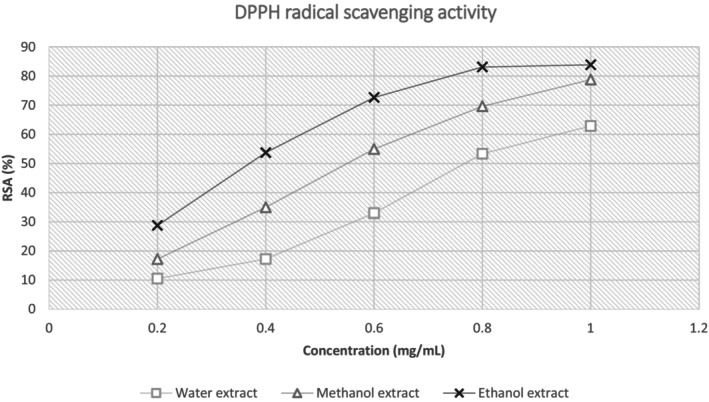
RSA of Liberica coffee silverskin in different solvents. Range of concentration tested 2–10 μg/mL. Ascorbic acid is the standard used as control.

The trend in RSA is plotted in Figure 5. From the graph plotted, the slope of the ethanol extract was steeper from concentration 0.2–0.8 mg/mL compared to the slope of methanol and water extract. The steepness of the slopes is significantly different (*p* < .05) between the alcohol and the aqueous extracts. This shows that the antioxidant ability of the extracts (methanol and ethanol) was much more rapid in comparison with the water extract. Based on the RSA graph in Figure 5, the plateau region of the ethanol extract showed earlier approximately at a concentration of 0.8 mg/mL compared to the methanol and water extracts in which both plateau regions did not appear in the graph even at a concentration of 1 mg/mL. The earlier onset of the plateau region indicates the maximum capacity of the extract to scavenge free radicals; thus, further increase in the concentration does not significantly increase the RSA. Earlier onset of the plateau region is also related to the steepness of the slope, for which, the earlier onset of both represents a higher RSA capacity of the extract against DPPH radicals (Nabavi et al., 2010).

Alluding from Table 2, it showed that the FRAP value of the CS sample is the highest when extracted with ethanol solvent with a value of 11.40 ± 18.57 μmol FSE/g. This was then followed by methanolic extract sample which was not that significant of a difference (*p* > 0.05) compared to the ethanolic extract with a value of 11.14 ± 17.83 μmol FSE/g, but still considered lower than the FRAP value by methanol. Lastly, aqueous extract with a value of 2.50 ± 3.98 μmol FSE/g. The *p*‐value (5%) is low, hence, the FRAP value is significantly different between extracts such that the difference in the type of solvent is notable in affecting the capability of the samples to reduce ferric into ferrous (Fe^2+^) ions.

Different standards used to calibrate will give different values of FRAP, such as shown in Benzie and Strain (1996) as they investigated different possible standards that be used in a standard compound for references and different standards showed different values of FRAP. This is also supported by the research completed by Ansori et al. (2021), as there was 24.41 ± 0.49 mg TE/g of FRAP value estimated from Liberica coffee ground spent. In the current study, the results of the Liberica CS showed only as high as 11.40 ± 18.57 μmol FSE/g of FRAP value when compared to standard FeSO_4_ instead of using Trolox. The results also show distinguishing difference of result as different parts were tested from the Liberica coffee plant. However, both current results and previous study show agreement in such that there is reduction of ferric, thus supporting the ability of the Liberica CS as a potential antioxidant agent.

## 4 CONCLUSION

In conclusion, this study has found that ethanol is an effective solvent for extracting bioactive compounds from Sarawak's Liberica CS and it is more recommended to use for extraction due to the higher phenolic and antioxidant compound extraction yield compared to methanol and water. Moreover, they are deemed to be less toxic compared to methanol and preferable for plant metabolite extraction based on the GC concept. For suggestion, further solvent conditions with different concentrations (v/v%) can be implemented on the Liberica CS, as multiple studies pointed out the effect of these concentrations on the levels of phenolic and antioxidant activities from the extracts. By identifying these compounds, Sarawak's adapted Liberica CS can be potentially utilized as a food ingredient other than be applied in cosmetic and health industries. Other than that, in agriculture, the CS can provide a new revenue stream for local coffee farmers, as well as coffee industries as the manipulation of by‐product can provide additional income while reducing the waste and impacts it may impose on the environment.

## AUTHOR CONTRIBUTIONS

**Nick Laurence Buyong:** Data curation (lead); formal analysis (lead); investigation (lead); writing – original draft (lead). **Elexson Nillian:** Conceptualization (lead); funding acquisition (lead); resources (supporting); supervision (lead); writing – review and editing (supporting).

## FUNDING INFORMATION

This research was funded by the Ministry of Higher Education Malaysia through the Fundamental Research Grant Scheme (FRGS) (FRGS/1/2019/STG05/UNIMAS/03/2) University Malaysia Sarawak, Kota Samarahan, Kuching, Sarawak.

## CONFLICT OF INTEREST STATEMENT

The authors declare that there is no conflict of interest in publishing the findings.

## ETHICAL REVIEW

This research does not involve human or animal testing.

## Data Availability

The data that support the findings of this research are available from the corresponding author upon reasonable request.
